# Myopia among children and adolescents: an epidemiological study in Fuzhou City

**DOI:** 10.3389/fped.2023.1161329

**Published:** 2023-06-13

**Authors:** Mei-hong Zhu, Tai-nan Lin, Jing-hua Lin, Qian Wen

**Affiliations:** ^1^ Department of Ophthalmology, Huaqiao University Hospital, Fujian, China; ^2^ Department of Ophthalmology, Fujian Provincial Governmental Hospital, Fujian, China

**Keywords:** adolescents, children, epidemiological, eyesight, investigation, myopia

## Abstract

**Objective:**

To provide a reference for the prevention and control of myopia by analyzing and discussing the findings of an epidemiological survey of the prevalence of myopia among children and adolescents in Fuzhou City from 2019 to 2021.

**Methods:**

Participants for this cross-sectional study were drawn from Gulou District and Minqing County in Fuzhou City using cluster random sampling to account for differences in population density, economic development, and other environmental variables.

**Results:**

Myopia was more prevalent in 2020 than in 2019, but by 2021 it had dropped to about the same level as in 2019. Myopia was more prevalent among girls than boys during the course of the study period, with a three-year prevalence of 44.72% for boys and 52.16% for girls. Mild myopia accounted for 24.14% of all cases, followed by moderate myopia at 19.62%, and severe myopia at 4.58%. Students in urban regions had a prevalence of myopia equivalent to that of students in the suburbs, and this prevalence rose with age.

**Conclusion:**

Myopia was quite prevalent among children and adolescents in Fuzhou City, and was shown to be steadily rising as students progressed through the school system. This suggests that all levels of government, educational institutions, medical facilities, and concerned parents in Fujian Province should focus on the issue of myopia and collaborate to reduce the risk factors for the development of myopia in school-aged participants.

## 1. Introduction

When the eye is at rest, the refractive system bends light rays so that they focus on the retina, but in the case of myopia, the parallel rays focus in front of the retina, preventing the formation of a sharp image (1). There is an increased risk of retinal detachment, macular degeneration, glaucoma, cataract, and even blindness in patients with myopia, especially severe myopia (2). Myopia can be slowed by time spent outside and/or exposure to bright light, possibly through dopamine-mediated pathways (3, 4). Myopia development is slowed in students who spend more time outside, and this is attributable to the sun's rays rather than physical exertion (5). There was a dramatic increase in the number of cases of myopia diagnosed in primary and secondary school students after the COVID-19 pandemic, with researchers attributing the rise to factors like excessive viewing of objects at close range and decreased participation in outdoor activities (6–8). Short-term studies conducted during the COVID-19 pandemic cannot accurately reflect the impact of the epidemic on myopia because myopia develops as the hyperopia reserve diopters decrease (9, 10).

In order to prevent severe myopia and preserve visual health, it is crucial to have a better understanding of the epidemiological features and influencing factors of myopia among children and adolescents (11, 12). Previous research indicates that the prevalence of myopia in this region is affected by economic conditions (13). Myopia is more prevalent among primary and secondary school children in coastal regions as well as in developed regions as opposed to underdeveloped regions. At the same time, the role that environmental factors play in causing myopia cannot be ignored.

A study by Rose suggested that chronically high levels of academic pressure contribute to a rise in the incidence of acquired myopia (14), while other studies demonstrate that a lack of outdoor exercise has a similar effect (15). We used a cross-sectional epidemiological approach and cluster sampling to identify our study participants, considering the current situation in China and Fuzhou as well as common diagnostic criteria for myopia in China.

## 2. Methods

### 2.1. Participants

The economic disparity between Fuzhou's coastal and upland regions is large. According to the United Nations Conference on Trade and Development (UNCTAD), we defined a region as economically developed if it had a per capita GDP above US$20,000; per capita GDP between US$8,000–US$20,000 is positioned as a medium-developed economic area; per capita GDP of less than US$8,000 is defined as an economically underdeveloped area. For this reason, in the first stage of the study, in October 2018, we used cluster random sampling to choose the prosperous Gulou District (per capita GDP was US$21,000) and the impoverished Minqing County (per capita GDP was US$7,080) from among the six districts and six counties in Fuzhou City. Additionally, we later randomly selected eight primary and secondary schools, including two senior high schools, two junior high schools, one primary school, and two kindergartens from these two places in 2019, 2020, and 2021.

We conducted an epidemiological investigation between January 2019 and December 2021, and students who volunteered to participate were enrolled. We excluded students with eye diseases other than refractive defects, such as cataract, glaucoma, and keratopathy. The number of students assessed each year was 4,622 in 2019, 4,250 in 2020, and 4,693 in 2021. Students who underwent vision correction with orthokeratology lenses were included in the study, their myopia level was assessed based on their diopter before using the orthokeratology lens and changes in their eye axis while wearing the lens.

The Ethics Committee of Fujian Provincial Office Hospital (2018–11) approved this study and confirmed that it was carried out in accordance with the principles of the Declaration of Helsinki. Before the study began, all students participating in the study, as well as their guardians, were fully informed of the study's objective, content, methods, and other aspects. The study was carried out with approval of the participating students and their parents, and informed consent was signed by the parents/guardians.

### 2.2. Research method

We utilized the international standard logarithmic visual acuity chart (LCD visual acuity chart, Tianjin Suowei) to assess uncorrected distance visual acuity (UDVA) in both eyes of the students, in school settings. During the eye examination, the students stood 5 meters from the light box, the visual acuity of both eyes (first right, then left) was tested, and the findings were recorded in decimal form. Eye drops were administered to each student, twice in each eye, 10 min apart. They were then asked to close their eyes and to rest for 20 min. After generating cycloplegia with cyclopentolate eye drops, we measured the diopter with an autorefractor (ARK-1a, Nidek, Japan). The test was done three times, and the results were averaged.

All procedures were performed by ophthalmologists, nurses, and technicians in line with standard operating protocols; all instruments were calibrated prior to the examination, and all examinations were conducted with the cooperation of the students. We chose the eye with higher equivalent spherical refraction in each student, for statistical analysis (16).

Before conducting the statistical analysis, decimal vision was converted to logarithm of the minimum angle of resolution (logMAR) vision. Spherical Equivalent Refraction (SER) = Diopter of Spherical Power (DS) + 1/2 Diopter of Cylindrical Power (DC). In this study, we classified myopia as monocular UDVA ≤ 0.8 and SER ≤ −0.50 D in computer optometry following cycloplegia (17), and students with myopia in one eye were defined as myopic. The myopic diopter was divided into three levels: mild myopia: UDVA ≤ 0.8, SER ≤ −0.50 D, and SER > −3.00 D; moderate myopia: UDVA ≤ 0.8, SER ≤ −3.00 D, and SER > −6.00 D; severe myopia: UDVA ≤ 0.8, SER ≤ −6.00 D.

### 2.3. Quality control

Examiners and equipment: All examiners were nationally certified as medical, technical, or nurse practitioners to ensure the authenticity and effectiveness of the data collected in this study. Prior to the study, all examiners underwent extensive professional training and were evaluated using a standardized system and form. They could only take part in the study if they passed the assessment and could determine the validity of the examination findings. The examiners were instructed to explain to the students the objective, meaning, and methodology of the examination and to gain their cooperation. The tools and equipment used in the examination were measured and calibrated on a regular basis, the visual acuity checklist met national requirements, and uniform instruments and equipment were supplied to each examination location.

### 2.4. Statistical analysis

Numerical data are expressed as the number of cases and percentage; measurement data with a normal distribution are expressed as mean ± standard deviation (*M* ± SD), and data that did not follow a normal distribution are expressed as median and interquartile range. The prevalence of myopia based on different variables was compared using the *χ*^2^ test or Fisher's exact test, and different myopic diopters were compared using ridit analysis. The prevalence of myopia in each variable was compared between genders using the Cochran–Mantel–Haenszel (CMH) test. The relationship between different ages and grades and prevalence of myopia was analyzed using linear regression, and the myopic prediction model was analyzed using stepwise logistic regression (forward likelihood ratio). The *P* value for inclusion and exclusion criteria were 0.05; *P* ≤ 0.05 indicated statistically significant differences.

## 3. Results

### 3.1. Gender distribution

**Figure 1:**
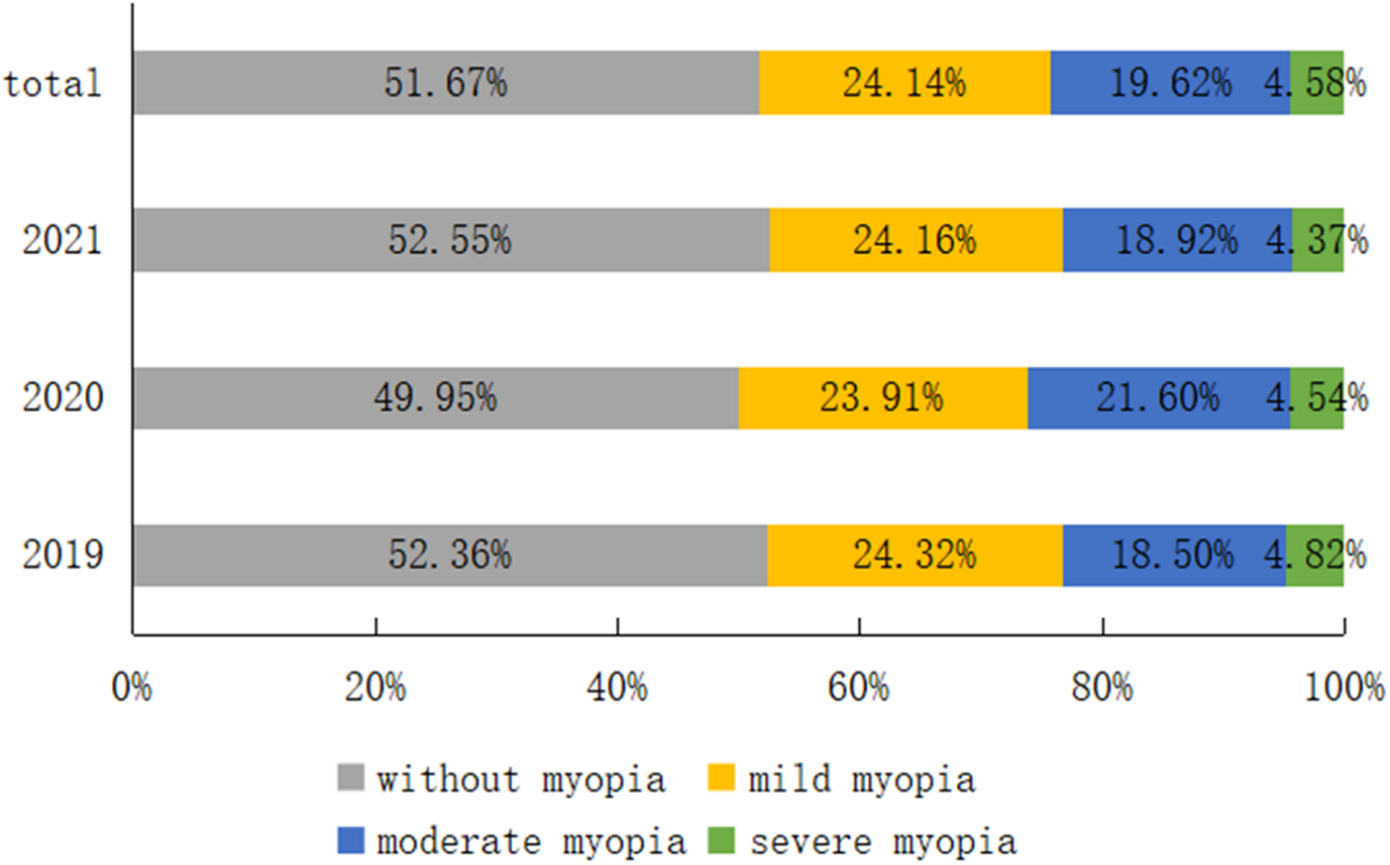
Proportion of various types of myopia in each year.

**Figure 2:**
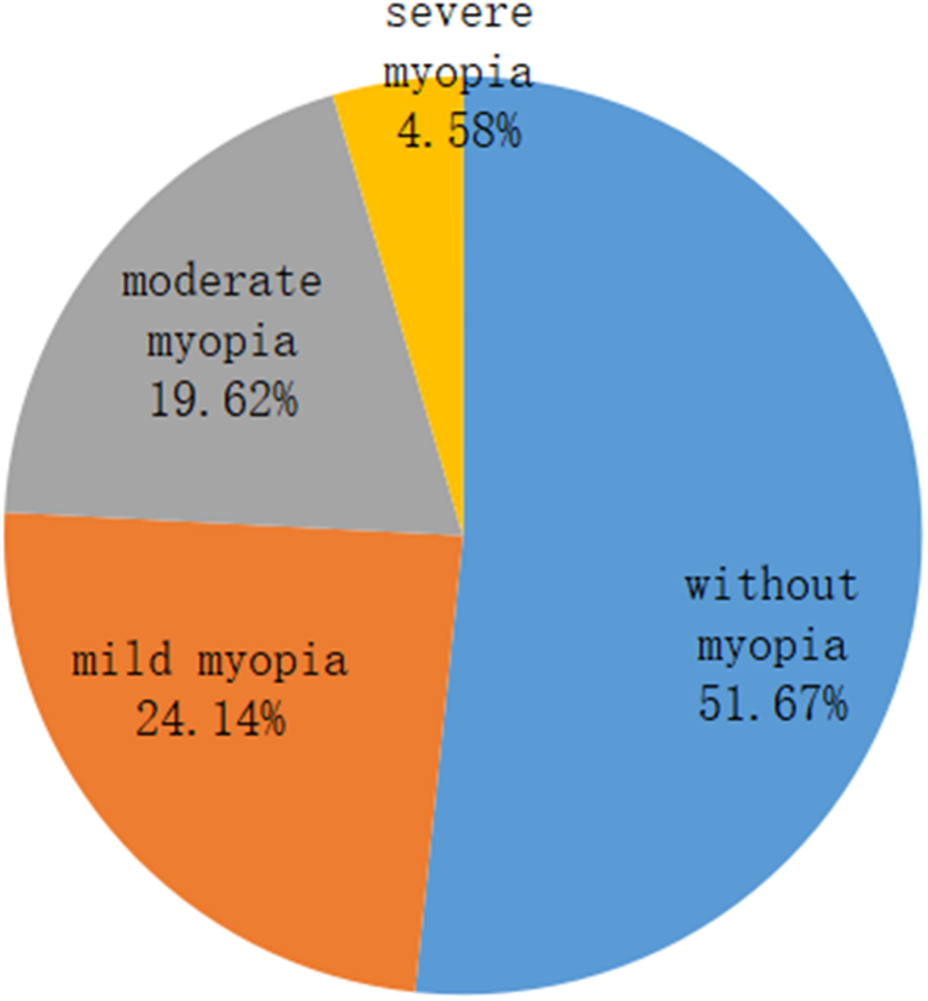
Proportion of various types of myopia.

There were 6,983 boys (51.48%) and 6,582 girls (48.52%) among the 13,565 primary and secondary school students. The prevalence of myopia was 44.72% among the boys and 52.16% among the girls. According to the myopia classification, mild myopia accounted for 24.14% followed by 19.62% with moderate myopia and 4.58% with severe myopia. The details are displayed in Figures 1, 2.

### 3.2. Age distribution

**Figure 3:**
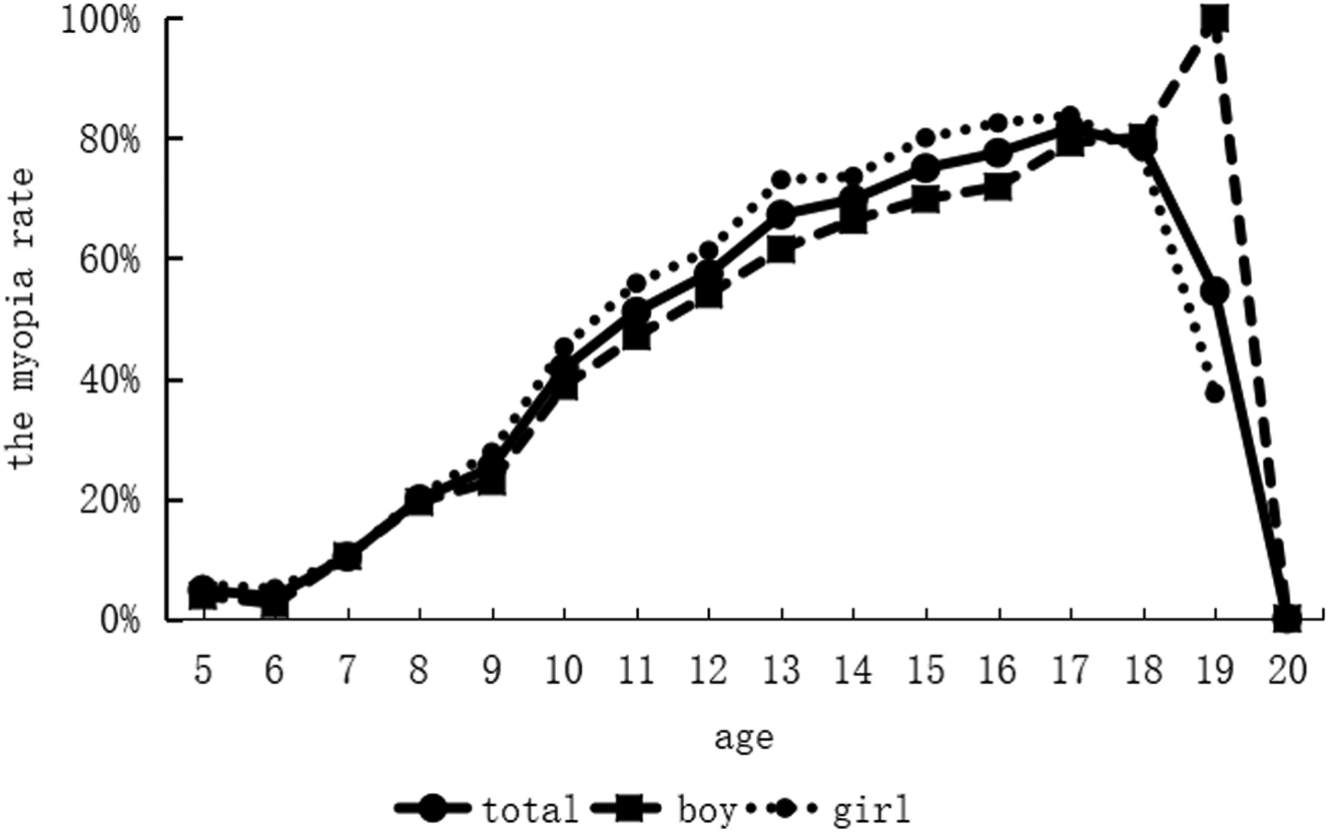
Variation of myopia prevalences among among children and adolescents at different ages.

Kindergarten students were 5–6 years old, primary school students were 7–12 years old, junior high school students were 13–15 years old, senior high school students were 16–18 years old, and vocational high school students were 16–20 years old. The maximum age was 20.37 years, the minimum age was 5.24 years, and the mean age was 11.89 years, with a standard deviation of 3.61 years. Among students without myopia, the maximum age was 20.37 years, the minimum age was 5.24 years, and the mean age was 10.04 years, with a standard deviation of 3.27 years. Among students with myopia, the maximum age was 19.47 years, the minimum age was 5.51 years, and the mean age was 13.87 years, with a standard deviation of 2.83 years. As seen in Figure 3, the prevalence of myopia increased in a nearly linear fashion with age, however, at the age of 20, the incidence of myopia plummeted suddenly since there was only one student who was not myopic.

### 3.3. Regional distribution

The participants were classified based on region into urban areas and suburban counties: 8,189 or 60.37% were from urban areas, with a prevalence of myopia of 48.93%, while 5,376 or 39.63% were from suburban counties, with a prevalence of myopia of 47.41%.

### 3.4. School and grade distribution

We examined students from kindergarten, primary school, junior high school, senior high school, and vocational high schools in this study, with most students (6,491) in primary school, with a prevalence of myopia of 28.33%, 3,253 students in junior high school, with a prevalence of myopia of 67.26%, 2,415 students in senior high school, with a prevalence of myopia of 82.36%, and 768 students in vocational high school, with a prevalence of myopia of 66.93%; the lowest number of students (638) was in kindergarten, with a prevalence of myopia of 4.08%.

In terms of age, the prevalence of myopia increased progressively as students progressed through the grades, peaking (up to 86.02%) in the third grade of senior high school. Although vocational high school is a senior high school, the academic pressure is not as intense as it is in senior high school. In this study, we discovered that the prevalence of myopia in vocational high school was equivalent to that in junior high school, but lower than that in senior high school for the same grade.

### 3.5. Comparison of variables

As shown in Table 1, the prevalence of myopia varied across primary and secondary school students of different years, genders, school types, ages, and grades, however there was no statistically significant difference in the prevalence of myopia among those in different regions. The findings of the pairwise comparison revealed that the prevalence of myopia in 2020 was higher than in 2019, but that the prevalence of myopia in 2021 was comparable to that of 2019, with girls having a higher prevalence than boys. According to the types of schools, the prevalence of myopia was highest in senior high school and lowest in kindergarten. According to the grade, the prevalence of myopia was highest in third grade of senior high school and lowest in senior kindergarten.

**Table 1:** Comparison of myopia prevalences as per various variables.

Variable		Without myopia	Myopia	Total	Myopia prevalence	*χ*^2^ value	*P* value
Year	2019	2,420	2,202	4,622	47.64%	7.338	0.026
	2020	2,123	2,127	4,250	50.05%		
	2021	2,466	2,227	4,693	47.45%		
Gender	Boy	3,860	3,123	6,983	44.72%	74.994	<0.001
	Girl	3,149	3,433	6,582	52.16%		
Region	Urban area	4,182	4,007	8,189	48.93%	2.991	0.084
	Suburban county	2,827	2,549	5,376	47.41%		
School type	Kindergarten	612	26	638	4.08%	3,233.068	<0.001
	Primary school	4,652	1,839	6,491	28.33%		
	Junior high school	1,065	2,188	3,253	67.26%		
	Vocational high school	254	514	768	66.93%		
	Senior high school	426	1,989	2,415	82.36%		
Age	5	260	13	273	4.76%	4,431.655	<0.001
	6	1,032	39	1,071	3.64%		
	7	949	109	1,058	10.30%		
	8	874	217	1,091	19.89%		
	9	802	270	1,072	25.19%		
	10	589	421	1,010	41.68%		
	11	565	591	1,156	51.12%		
	12	468	631	1,099	57.42%		
	13	362	744	1,106	67.27%		
	14	322	745	1,067	69.82%		
	15	283	850	1,133	75.02%		
	16	242	837	1,079	77.57%		
	17	187	829	1,016	81.59%		
	18	68	254	322	78.88%		
	19	5	6	11	54.55%		
	20	1	0	1	0.00%		
Grade	Top class in kindergarten	612	26	638	4.08%	4,070.075	<0.001
	First grade in primary school	1,042	48	1,090	4.40%		
	Second grade in primary school	936	143	1,079	13.25%		
	Third grade in primary school	842	229	1,071	21.38%		
	Fourth grade in primary school	724	332	1,056	31.44%		
	Fifth grade in primary school	591	480	1,071	44.82%		
	Sixth grade in primary school	517	607	1,124	54.00%		
	First grade in junior high school	433	653	1,086	60.13%		
	Second grade in junior high school	334	755	1,089	69.33%		
	Third grade in junior high school	298	780	1,078	72.36%		
	First grade in senior high school	171	653	824	79.25%		
	Second grade in senior high school	145	659	804	81.97%		
	Third grade in senior high school	110	677	787	86.02%		
	First grade in vocational high school	97	155	252	61.51%		
	Second grade in vocational high school	88	165	253	65.22%		
	Third grade in vocational high school	69	194	263	73.76%		

The prevalence of myopia increased continuously as students became older, however it decreased between the ages of 19 and 20 years as there were few students at this age in this study. We performed linear regression analysis with age as the independent variable and prevalence of myopia as the dependent variable. The results revealed that there was a linear correlation between age and the prevalence of myopia, *r* = 0.585; the correlation coefficient was not high as there were fewer senior school students aged 19 and 20 years in the study; the linear equation was: Prevalence of myopia = 0.037 × age − 0.010 (*F* = 7.272, *P* = 0.017). After excluding students aged 19 and 20 years, we repeated the correlation and regression analyses, *r* = 0.975; the correlation coefficient was high, and the linear equation was: Prevalence of myopia = 0.069 × age − 0.314 (*F* = 233.321, *P* < 0.001).

We performed linear regression analysis with grade as the independent variable (the value was 1 for kindergarten, 2 for first grade, and so on; the value for first grade in vocational high school was the intermediate value of the third grade in junior high school and the first grade in senior high school as the academic pressure was less severe than that in the first grade in senior high school, and so on) and the prevalence of myopia as the dependent variable; the results revealed that there was a linear correlation between grade and the prevalence of myopia, *r* = 0.969; the linear equation was: Prevalence of myopia = 0.069 × grade − 0.026 (*F* = 216.306, *P* < 0.001).

Due to the significant difference in the prevalence of myopia between boys and girls, we performed the CMH test for each variable with gender as the block factor; the results are shown in Tables 2, 3. As shown in Table 2, the prevalence of myopia in girls was higher than that in boys for all variables; additionally, as shown in Table 3, except for the year and region, there were significant differences in the prevalence of myopia after gender stratification for other variables.

**Table 2:** Comparison of myopia prevalences among boys and girls for each variable.

Variable		Male				Female			
		Non-myopia	Myopia	Total	Myopia prevalence	Non-myopia	Myopia	Total	Myopia prevalence
Year	2019	1,299	1,069	2,368	45.14%	1,121	1,133	2,254	50.27%
	2020	1,187	1,005	2,192	45.85%	936	1,122	2,058	54.52%
	2021	1,374	1,049	2,423	43.29%	1,092	1,178	2,270	51.89%
Region	Urban area	2,316	1,841	4,157	44.29%	1,866	2,166	4,032	53.72%
	Suburban county	1,544	1,282	2,826	45.36%	1,283	1,267	2,550	49.69%
School type	Kindergarten	310	9	319	2.82%	302	17	319	5.33%
	Primary school	2,542	917	3,459	26.51%	2,110	922	3,032	30.41%
	Junior high school	634	1,058	1,692	62.53%	431	1,130	1,561	72.39%
	Senior high school	277	1,007	1,284	78.43%	149	982	1,131	86.83%
	Vocational high school	97	132	229	57.64%	157	382	539	70.87%
Age	5	126	5	131	3.82%	134	8	142	5.63%
	6	557	14	571	2.45%	475	25	500	5.00%
	7	500	58	558	10.39%	449	51	500	10.20%
	8	468	113	581	19.45%	406	104	510	20.39%
	9	429	127	556	22.84%	373	143	516	27.71%
	10	337	213	550	38.73%	252	208	460	45.22%
	11	327	290	617	47.00%	238	301	539	55.84%
	12	269	316	585	54.02%	199	315	514	61.28%
	13	214	342	556	61.51%	148	402	550	73.09%
	14	188	371	559	66.37%	134	374	508	73.62%
	15	169	392	561	69.88%	114	458	572	80.07%
	16	142	364	506	71.94%	100	473	573	82.55%
	17	102	390	492	79.27%	85	439	524	83.78%
	18	31	125	156	80.13%	37	129	166	77.71%
	19	0	3	3	100.00%	5	3	8	37.50%
	20	1	0	1	0.00%				
Grade	Senior kindergarten	310	9	319	2.82%	302	17	319	5.33%
	First grade in primary school	560	20	580	3.45%	482	28	510	5.49%
	Second grade in primary school	502	78	580	13.45%	434	65	499	13.03%
	Third grade in primary school	448	120	568	21.13%	394	109	503	21.67%
	Fourth grade in primary school	395	166	561	29.59%	329	166	495	33.54%
	Fifth grade in primary school	344	228	572	39.86%	247	252	499	50.50%
	Sixth grade in primary school	293	305	598	51.00%	224	302	526	57.41%
	First grade in junior high school	252	315	567	55.56%	181	338	519	65.13%
	Second grade in junior high school	192	355	547	64.90%	142	400	542	73.80%
	Third grade in junior high school	190	388	578	67.13%	108	392	500	78.40%
	First grade in senior high school	119	315	434	72.58%	52	338	390	86.67%
	Second grade in senior high school	91	311	402	77.36%	54	348	402	86.57%
	Third grade in senior high school	67	381	448	85.04%	43	296	339	87.32%
	First grade in vocational high school	34	38	72	52.78%	63	117	180	65.00%
	Second grade in vocational high school	48	53	101	52.48%	40	112	152	73.68%
	Third grade in vocational high school	15	41	56	73.21%	54	153	207	73.91%

**Table 3:** CMH test results of myopia prevalence among boys and girls for each variable.

Variable	Female		Male		Total	
	*χ*^2^ value	*P* value	*χ*^2^ value	*P* value	*χ*^2^ value	*P* value
Year	7.893	0.019	3.296	0.192	7.338	0.026
Gender	10.187	0.001	0.790	0.374	2.991	0.084
Region	1,731.523	<0.001	1,513.124	<0.001	3,233.068	<0.001
School type	2,358.272	<0.001	2,091.648	<0.001	4,431.655	<0.001
Grade	2,193.078	<0.001	1,911.831	<0.001	4,070.075	<0.001

We did a logistic analysis with myopia set as 1 and non-myopia set as 0 as the independent variable, and the year, urban area, grade, school type, gender, and age as the dependent variables (there was collinearity between age and grade, and analysis showed that the results were more realistic when selecting age as the independent variable), and the results are shown in Table 4.

**Table 4:** Results of univariate logistic regression analysis.

Variable	*β*	S.E.	Wald	df	*P* value	OR	95% OR CI	
							Lower	Upper
Year			1.277	2	0.528			
Year(1)	−0.046	0.050	0.840	1	0.360	0.955	0.867	1.053
Year(2)	−0.052	0.050	1.053	1	0.305	0.950	0.860	1.048
Region	−0.347	0.042	66.824	1	<0.001	0.707	0.651	0.768
Gender	−0.326	0.041	63.247	1	<0.001	0.722	0.666	0.782
Age	0.443	0.015	816.779	1	<0.001	1.557	1.510	1.605
Constant	−4.032	0.111	1,311.759	1	<0.001	0.018		

As shown in Table 5, there were statistically significant differences in the prevalence of myopia in different years and genders, wherein the proportion of moderate myopia in 2020 was significantly higher than that in 2019 and 2021, and there was no statistically significant difference in other distributions; the myopia diopter in girls was higher than that in boys.

**Table 5:** Myopia diopters in different years and genders.

Variable		Without myopia	Mild myopia	Moderate myopia	Severe myopia	Hc value	*P* value
Year	2019	2,420	1,124	855	223	10.217	<0.001
	2020	2,123	1,016	918	193		
	2021	2,466	1,134	888	205		
Gender	Boy	3,860	1,524	1,311	288	59.787	<0.001
	Girl	3,149	1,750	1,350	333		

## 4. Discussion

Myopia has become a serious public health issue in China in recent years, with an increase in prevalence among children and adolescents (18). With the rapid social and economic growth in China since 2010, the amount of time spent watching digital displays from a close distance has dramatically increased due to the popularity of electronic items, which may be associated with a higher prevalence of myopia among students (19, 20). According to World Health Organization (WHO) data, students in more than 150 countries and regions had to study at home in 2020 due to COVID-19 prevention and control measures, and a large number of children and adolescents continued to study at home in 2021, when the number of cases in these countries and regions decreased slightly.

Previous research has revealed that since the outbreak of the COVID-19 epidemic, students have been studying online at home (21) while their outside activities have been restricted (22), resulting in an increased prevalence of myopia, particularly among minors (23, 24). This shows that children and adolescents require regular and continuous visual acuity testing. It is beneficial to utilize cyclopentolate eye drops, which have been recommended in international epidemiological studies, for inducing cycloplegia during eye examinations (25). When compared to non-cycloplegic refraction, cycloplegic refraction can more accurately reflect the true condition of myopia in children and adolescents (26). In this study, an epidemiological assessment of myopia among children and adolescents in Gulou District and Minqing County, Fuzhou City, was carried out over three years, from 2019 to 2021.

The results of this study show that the incidence of myopia in 2020 was greater than in 2019 and 2021 was similar to the levels in 2019, however there was some variation from year to year. One possible explanation is that during the severe epidemic in 2020, children and adolescents in Fuzhou were isolated at home, and the increased prevalence of myopia was related to their inability to go outdoors, reduced outdoor exercise, and use of digital displays and electronic devices for study. Our findings are consistent with the results of Chen and Cai (27, 28) that decreased outdoor exercise and increased use of digital displays and electronic products increased the incidence of myopia.

Prior studies have found that the proportion of Chinese students with moderate and severe myopia rose as the incidence of myopia increased (18, 29). According to our findings, the proportion of children and adolescents with moderate myopia in 2020 was significantly higher than in 2019 and 2021, which could be attributed to the progression of mild myopia into moderate myopia in some children and adolescents with the increase in myopic diopters in 2020. At the same time, the incidence of myopia was significantly lower in 2021 than in 2020, with the difference being statistically significant, and it was on par with 2019. Possible causes include children spending more time outdoors and using fewer electronic devices as the severity of the pandemic waned. At the same time, schools and parents placed more emphasis on myopia in students (30), and low-concentration atropine eye drops were extensively prescribed in China to prevent myopia and postpone its progression (31, 32), lowering the incidence of myopia to the level seen in 2019. However, some school-age children and adolescents chosen as participants in this study were not included after they graduated, and more research is needed to examine the long-term impact of the epidemic on the incidence of myopia.

The findings of this study indicate that the prevalence of myopia among girls was higher than that among boys from 2019 to 2021, which is consistent with the findings of earlier studies (29, 33). Possible explanations include gender-specific differences in puberty and sleep habits, as well as the effects of increased screen time and decreased physical activity among girls. Myopia has been shown to be equally prevalent in adult males and females in other studies; thus, more studies are needed to confirm this finding. While girls and boys had the same hyperopia reserve levels, the cumulative incidence of myopia among the former was significantly higher than that among the latter, indicating that it is critical for girls to protect their hyperopia reserve and spend more time outdoors.

The results of this study also revealed that based on school type, the prevalence of myopia could be ranked as senior high school > junior high school = vocational high school > primary school > kindergarten; based on grade, the prevalence of myopia also increased with age, which is directly related to the academic pressure faced by students. Our findings however, differed from previous studies (34, 35), which found that the prevalence of myopia among children and adolescents in urban areas was higher than that in rural areas. In our study, we found that the prevalence of myopia in urban and suburban students was nearly identical. The prevalence of myopia was 48.93% in urban areas and 47.41% in suburban counties, with no statistically significant difference (the results are shown in Table 1). This is due in large part to the fact that the economic gap between urban and rural areas in China has shrunk in recent years, and parents in the suburbs have begun to pay attention to education, which has led to more time spent by students engaged in viewing digital displays from a close distance and less time spent engaging in outdoor exercise.

In this study, we performed a cross-sectional survey of students in Fuzhou City for the first time over the three years of the pandemic, to explore the prevalence of myopia. The use of a scientific sampling procedure, as well as the large sample size and generally comprehensive data acquired, ensured that the study results were representative to some extent. Furthermore, we strictly ensured that the examination procedures followed national standards, and we used scientific criteria for diagnosis and exclusion, which allowed us to compare the results of this survey to other studies in China.

## 5. Limitations

First, it is possible that some students with myopia who were nervous about the checkup were not included during the initial three-year screening for this study, leading to an underestimate of the true prevalence of myopia and severe myopia. Second, due to a lack of information, some students with undiagnosed eye problems may have been overlooked and omitted from the study. However, given the large sample size in this study, this limitation is likely to have had little impact on the findings. Finally, another limitation of this study is the lack of assessment of risk factors associated with myopia.

## 6. Conclusion

In conclusion, the prevalence of myopia in 2020 was higher than in 2019, but the prevalence of myopia in 2021 was comparable to that of 2019 in Fuzhou City. The prevalence of myopia among girls was higher than that among boys in each year; during the three years, the prevalence of myopia was 44.72% among boys and 52.16% among girls. In terms of myopia type, mild myopia was predominant, accounting for 24.14%, followed by 19.62% with moderate myopia and 4.58% with severe myopia, indicating that the incidence of myopia was high. The government, schools, hospitals, and parents in Fujian Province must collaborate to reduce myopia and other preventable eye conditions among children and teenagers.

## Data Availability

The original contributions presented in the study are included in the article, further inquiries can be directed to the corresponding author.
